# The Importance of Cancer-Related and Other Health Factors on Cognition Among Older-Adult Long-Term Survivors

**DOI:** 10.1093/geroni/igaa057.1398

**Published:** 2020-12-16

**Authors:** Gabrielle Beck, Gary Deimling, Boaz Kahana, Eva Kahana, Erin Phelps, Spencier Ciaralli

**Affiliations:** 1 Case Western Reserve University, Cleveland, Ohio, United States; 2 Cleveland State University, Cleveland, Ohio, United States

## Abstract

Previous research has identified cancer and cancer-treatment related effects on survivors’ mental impairment including memory and concentration. However, research has not systematically examined the relative impact of cancer in the context of age and other age-related health challenges common in later life. This paper compares the effects of cancer-related factors with other health challenges faced by 471 older adult long-term survivors from an NCI-funded study of a randomly selected tumor registry sample from a major comprehensive cancer center. Having had chemotherapy is associated with several cognitive outcomes including memory and concentration. Survivors who reported more cancer-related symptoms during treatment reported a greater number of cognitive symptoms even decades after treatment. Importantly, other comorbid health problems as well as social factors were found to be important in explaining symptoms of cognitive impairment in this older adult sample. These findings suggest that health care and mental health providers consider the range of health challenges, including those related to cancer and its treatment, as they provide patient centered care.

